# Customized Regulation of Diverse Stress Response Genes by the Multiple Antibiotic Resistance Activator MarA

**DOI:** 10.1371/journal.pcbi.1005310

**Published:** 2017-01-06

**Authors:** Nicholas A. Rossi, Mary J. Dunlop

**Affiliations:** 1 Molecular Biology, Cell Biology & Biochemistry Program, Boston University, Boston, MA United States of America; 2 Department of Biomedical Engineering, Boston University, Boston, MA United States of America; Ottawa University, CANADA

## Abstract

Stress response networks frequently have a single upstream regulator that controls many downstream genes. However, the downstream targets are often diverse, therefore it remains unclear how their expression is specialized when under the command of a common regulator. To address this, we focused on a stress response network where the multiple antibiotic resistance activator MarA from *Escherichia coli* regulates diverse targets ranging from small RNAs to efflux pumps. Using single-cell experiments and computational modeling, we showed that each downstream gene studied has distinct activation, noise, and information transmission properties. Critically, our results demonstrate that understanding biological context is essential; we found examples where strong activation only occurs outside physiologically relevant ranges of MarA and others where noise is high at wild type MarA levels and decreases as MarA reaches its physiological limit. These results demonstrate how a single regulatory protein can maintain specificity while orchestrating the response of many downstream genes.

## Author Summary

Bacteria can sense and respond to stress in their environment. This process is often coordinated by a master regulator that turns on or off many downstream genes, allowing the cell to survive the stress. However, individual genes encode products that are diverse and optimal expression for each gene may differ. Here, we focus on how expression of diverse downstream genes is optimized by targets of the multiple antibiotic resistance activator MarA. Using single-cell experiments and computational modeling we show that downstream genes process MarA signals differently, with unique activation, noise, and information transmission properties. We find that each downstream gene’s response depends critically on the level of the input MarA. Furthermore, by swapping parts of the regulatory elements of genes we were able to create novel responses. This suggests that these properties can be readily tuned by evolution. Our findings show how a network with diverse downstream genes can be used to process the same command to achieve many distinct outputs, which work together to coordinate the response to stress.

## Introduction

Genetic networks often feature master regulators that control suites of downstream genes. This type of architecture is particularly common in stress response; examples include Msn2 and Crz1 in *Saccharomyces cerevisiae*, σ^B^ in *Bacillus subtillis*, and the multiple antibiotic resistance activator MarA in *Escherichia coli* [1–4]. Each of these regulators controls tens to hundreds of diverse downstream targets with widely varying functional roles. For example, MarA regulates small RNAs, metabolic enzymes, efflux pumps, and regulatory proteins [1,5]. This diversity of gene products raises two questions: First, how is one signal from an upstream regulator decoded differently by multiple downstream targets? Second, what are the potential benefits and tradeoffs that define how these genes respond? To address this, we examined three key properties: activation, transmitted noise, and transmitted information. Although the properties are linked, they can be adjusted in a variety of ways to tailor the response of individual genes.

Activation is set by molecular details of the transcription factor and the DNA to which it binds. The resulting transfer function is often described by the sigmoidal Hill function [6]. Here we focus on MarA, which regulates over 60 downstream targets by binding to a well-characterized degenerate binding sequence in their promoters known as the ‘marbox’ [5,7]. Previous studies have mapped the activation properties of genes controlled by MarA, finding that the amount of MarA needed to turn on expression varies greatly among its targets, with a 19-fold difference in the dissociation constant (K_d_) between genes [1]. The majority of downstream genes are only weakly activated unless MarA is overexpressed far beyond physiologically relevant levels [1]. This is due to the high dissociation constants of the downstream promoters [8], and provokes the question of why these genes are regulated by MarA at all.

Noise provides insight into why this may be the case. Recent studies at the single-cell level suggest that although most cells only express low levels of MarA, levels are high in a small subset of the population due to cell-to-cell differences in gene expression [9,10]. These single-cell differences allow a subpopulation of cells to survive antibiotic exposure, and survivors can recolonize after the stress has passed [9]. Therefore, noise in MarA may turn on expression of costly downstream genes only in a small subset of the population to hedge against future uncertainty. This role for noise in stress response is observed frequently, such as in sporulation and competence in *B*. *subtilis*, which allow subpopulations of cells to survive periods of extreme stress [11–13]. When a noisy input controls downstream targets that have high dissociation constants, low-level fluctuations can be filtered, while larger signals are transmitted [14,15].

The relationship between the level of the input and the ability to activate downstream genes defines whether information is transmitted between input and output. This can be quantified using the metric channel capacity, which is the theoretical limit of how well information can be passed through a network (similar to the diameter of a pipe, or channel) [16,17]. Channel capacity depends both on the bounds of possible input values for the natural system and on the shape of the activation curve [17–19]. For instance, if the realistic range of MarA values are constrained such that a downstream gene is only weakly activated, then the channel capacity is low. As the range of MarA values widens, the channel capacity may either increase, if there is a corresponding increase in downstream gene expression, or remain low, if expression does not change, as in the case where the response is saturated. Therefore the ability to transmit information depends on both the input distribution and where these values fall on the activation curve.

Expression of individual downstream genes can be tailored to balance activation, noise, and information transmission in any number of ways. For instance, certain genetic regulatory elements transmit information near the channel capacity [20,21], while others lose considerable information due to noise or active filtering [22,23]. Therefore, gene regulation may be tailored to balance these properties.

Using MarA and its downstream genes as a case study in multi-gene regulation, we investigated how expression is tailored at the single-cell level. We identified two qualitative classes of promoters: amplifying and filtering. The amplifying promoters generate diversity and have a high channel capacity at low levels of MarA. In contrast, the filtering promoters have low noise and only transmit information when MarA is high. These qualitative differences in promoter classes correlate with the functional differences in the gene products they control. We created chimeric promoters by swapping the MarA binding sequences between downstream promoters from these groups and easily altered their quantitative characteristics. Therefore, this binding sequence can serve as an evolutionary target for tuning output response.

## Results

We first asked how individual genes respond to MarA at the single-cell level. To do this, we transformed *E*. *coli* MG1655 Δ*marRAB* with a plasmid bearing an IPTG-inducible version of *marA* transcriptionally-fused to red fluorescent protein (*rfp*). We cotransformed cells containing the inducible *marA-rfp* plasmid with a second plasmid containing green fluorescent protein (*gfp*) linked to a promoter for a MarA-controlled downstream gene. We then simultaneously measured RFP and GFP levels in individual cells using microscopy. By adding IPTG, we increased MarA (measured by RFP), which activated expression of the downstream promoter (measured by GFP). IPTG induction allowed us to capture a broad range of MarA levels (S1 Fig). These data provide single-cell resolution measurements of downstream gene expression as a function of MarA.

**Fig 1:**
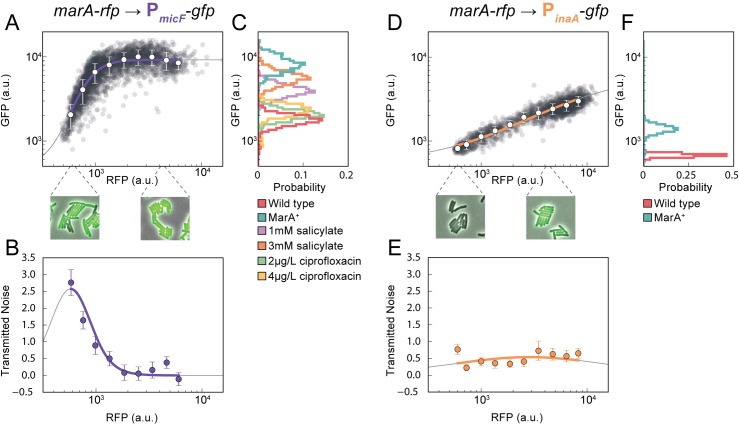
Downstream genes *micF* and *inaA* have distinct noise and activation profiles over biologically relevant ranges of MarA. **(A)** Activation of P_*micF*_ at the single-cell level using inducible MarA. Each gray dot corresponds to a single cell with data from snapshots under multiple IPTG concentrations (S1 Fig); white dots are means with standard deviations from binned data. Purple line is a Hill function fit to the mean values within the experimentally measured range; gray line is the function outside this range. Images show two example snapshots under low and high activation using 0 and 500 μM IPTG. **(B)** Transmitted noise through P_*micF*_. Transmitted noise is calculated from experimental data using Eq 2; error bars are the standard deviation determined by bootstrap resampling (Methods). Solid lines are analytical solutions. **(C)** P_*micF*_ expression in reporter-only experiments using wild type and MarA^+^ genetic backgrounds, and with salicylate and ciprofloxacin chemical stresses. **(D)** Single cell P_*inaA*_ activation as a function of MarA. Statistics are calculated as in (A). Note that the images show little change in cellular diversity as MarA increases. **(E)** Transmitted noise for P_*inaA*_. Analytical solutions and experimental data are as in (B). **(F)** P_*inaA*_ expression in wild type and MarA^+^ genetic backgrounds.

Initially, we quantified expression from the promoter P_*micF*_ in response to MarA. *micF* encodes a small RNA that represses the outer membrane porin OmpF, decreasing vulnerability to a number of stressors including antibiotics and osmotic shock [24]. Previous population-level analysis of P_*micF*_ has indicated that the promoter has a relatively low K_d_, suggesting that it should turn on with low levels of MarA [1]. Consistent with this, P_*micF*_ expression increased in response to MarA induction and eventually saturated (Fig 1A). We eliminated the possibility that this result was due to artificial crosstalk between the RFP and GFP channels by constructing a control strain where the P_*micF*_ reporter was cotransformed with a plasmid with inducible *rfp* and no *marA* and observed no spurious crosstalk effects (S2 Fig).

Consistent with previous work on noise propagation [25], we noted that noise in GFP expression changed along the P_*micF*_ activation curve. Diversity in single-cell expression is highest at low levels of MarA. To quantify this effect, we calculated the normalized coefficient of variation as a measure of transmitted noise (Fig 1B). Analytically, this value is proportional to the local slope of the activation curve [19]. In other words, transmitted noise is highest where the Hill function is the steepest.

To investigate where on this curve physiological levels of MarA fall, we conducted experiments using the P_*micF*_ reporter in two genetic backgrounds. First, we measured the lower bound of the P_*micF*_ response using wild type *E*. *coli* MG1655. These represent the natural levels of P_*micF*_ under unstressed conditions with basal MarA expression. Second, we measured the upper bound of the P_*micF*_ response by using a strain we denote MarA^+^, where the chromosomal copies of both MarR binding sites are inactivated. The *marRAB* operon is induced when repression by MarR is inhibited, therefore by inactivating these repressor binding sites this strain expresses the maximum physiologically realistic level of MarA. Measurements from these two strains have distinctly different levels of P_*micF*_ reporter expression (Fig 1C).

To confirm that these bounds on the physiological levels of MarA were appropriate we also subjected wild type cells to several chemical stresses. We first used salicylate, the canonical inducer of the *marRAB* operon, which causes a conformational change in MarR that prevents it from repressing *marRAB* expression [26]. Using 1 and 3 mM salicylate, we found expression to fall within the upper and lower bounds established by the wild type and MarA^+^ strains. Additionally, the quinolone ciprofloxacin can indirectly inhibit MarR by increasing intracellular copper levels [26]. Exposure to sublethal levels of ciprofloxacin (2 and 4 μg/L) also resulted in intermediate levels of P_*micF*_ expression. Together, these results outline the biologically relevant range of P_*micF*_ expression.

Although P_*micF*_ expression changes dramatically as MarA sweeps across physiologically relevant values, bulk measurements of other MarA-activated genes have suggested that many MarA-regulated genes are not strongly activated [1]. Thus, we next tested expression of P_*inaA*_, which has a K_d_ ten times higher than P_*micF*_ [1]. While the exact role of *inaA* is unknown, it is a pH-inducible gene involved in stress response [27]. In sharp contrast to P_*micF*_, P_*inaA*_ shows a gradual response to MarA, which never saturates over the tested range (Fig 1D). We quantified transmitted noise and our results show good agreement with the theoretical prediction based on the slope of the fitted Hill function (Fig 1E). In contrast to P_*micF*_, P_*inaA*_ noise levels remain low across all MarA values. Paralleling the experiments with P_*micF*_, we used the wild type and MarA^+^ genetic backgrounds to establish the physiologically relevant upper and lower bounds for P_*inaA*_ expression (Fig 1F). The two distributions are noticeably closer together than in the case of P_*micF*_, which is expected given the gradual slope of the Hill function.

Our initial results with P_*micF*_ and P_*inaA*_ reveal that even with a common upstream regulator, there can be categorical differences in downstream gene expression. These differences include population-level response characteristics such as the shape of the activation curve and also single-cell level effects such as variability in gene expression. These effects are related since transmitted noise depends on the slope of the activation curve.

**Fig 2:**
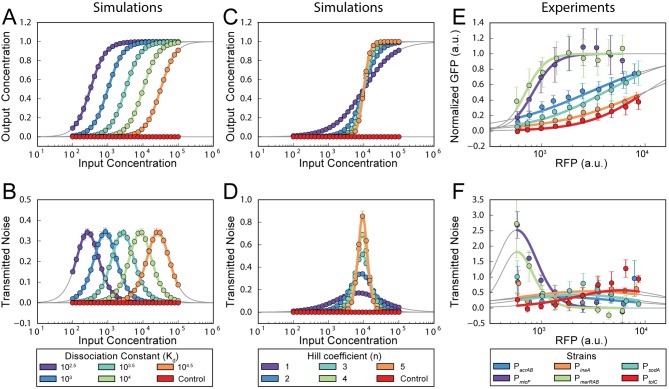
Dissociation constant and Hill coefficient are critical for determining how different downstream genes interpret the same input. **(A)** Stochastic simulation (dots) and analytical solutions (lines) for five downstream genes with a range of dissociation constants, K_d_. The control (red) does not depend on the input. Error bars are standard deviations of binned data, but are too small to be visible. **(B)** Transmitted noise as a function of K_d_. Dots are mean of computational results; solid lines are analytical solutions. Transmitted noise is calculated using Eq 2; error bars are standard deviation from bootstrap resampling. **(C)** Stochastic simulation (dots) and analytical solutions (lines) for five downstream genes with a range of Hill coefficients, n, and an unregulated control (red). Error bars show standard deviations of binned data. **(D)** Transmitted noise as a function of n. Error bars as in (B). **(E)** Experimental results showing activation curves of six MarA-regulated genes. Error bars show standard deviations of binned data. Lines show analytical solutions. **(F)** Transmitted noise curves for the six downstream genes. Error bars are standard deviation from bootstrap resampling.

To explore how different characteristics of downstream gene activation influence transmitted noise, we used a computational model to simulate systems with different values for the dissociation constant (K_d_) and Hill coefficient (n). We simulated a noisy activator regulating five downstream genes with different K_d_ values (Fig 2A). In addition, we included a control not regulated by the activator. We then calculated the transmitted noise for each, and the mean values of our stochastic simulations show excellent agreement with our analytical solutions (Fig 2B).

Given the sigmoidal shape of the Hill function, K_d_ alone can determine whether a promoter filters or amplifies a given input signal. Moreover, K_d_ is related to the strength of binding between the transcription factor and its associated binding site. This parameter is easily altered by mutations in the binding site and is therefore a potential evolutionary tuning knob. Indeed, the marbox has substantial variation among the myriad of genes regulated by MarA [8].

In contrast to K_d_, altering the Hill coefficient n primarily affects the magnitude and shape of the noise response (Fig 2C and 2D). Genes with high n values have high transmitted noise over narrow input ranges, while lower n values correspond to lower, broader responses. We note that other parameters, such as activation and degradation rates, can also influence transmitted noise (Supplementary Information and S3 Fig). In general terms, K_d_ controls the activator levels where the transmitted noise is highest, while n primarily affects the magnitude of the transmitted noise.

We next asked whether the activation and transmitted noise profiles of a diverse set of MarA-regulated genes varied as a function of n and K_d_ as in our computational simulation. We expanded our single-cell studies to include a total of six MarA-regulated promoters: P_*micF*_ and P_*inaA*_ discussed previously, and the promoters for efflux pump genes P_*acrAB*_ and P_*tolC*_, superoxide dismutase P_*sodA*_, and P_*marRAB*_. We selected these genes based on their diverse responses to MarA at the population level [1]. For each, we used IPTG-inducible MarA and measured activation and transmitted noise in the downstream promoters (Fig 2E and 2F). Of the six genes we measured, we observed a range of expression profiles that fall broadly into two groups. First, the ‘amplifying’ group, which includes P_*micF*_ and P_*marRAB*_, saturates over the examined range of MarA inputs. These promoters have lower K_d_ values and larger n values. For these genes, low-level fluctuations in MarA will become large fluctuations in the downstream gene. In contrast, the ‘filtering’ group includes P_*acrAB*_, P_*inaA*_, P_*sodA*_, and P_*tolC*_. These genes do not saturate over the MarA range we tested and have lower n values. In this group, low-level fluctuations in MarA are filtered in the downstream gene, attenuating both the signal and the noise. These two classes of genes have categorically different transmitted noise profiles (Fig 2F). The amplifying genes have high transmitted noise peaks at low levels of MarA and drop sharply as MarA increases, while the filtering genes have low, broad transmitted noise curves.

**Fig 3:**
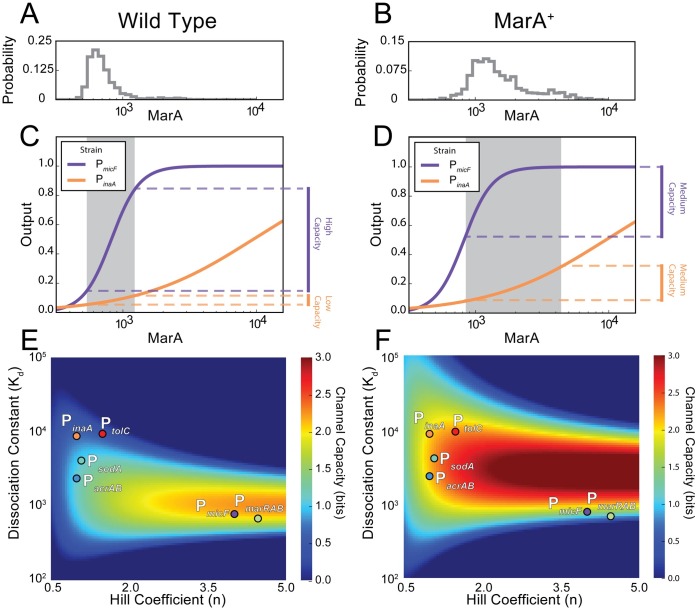
Channel capacity for downstream genes over biologically relevant ranges of MarA. **(A)** Histogram of the estimated MarA levels in wild type cells (S4 Fig). **(B)** Estimated MarA levels in MarA^+^ cells. **(C)** Schematic showing how wild type MarA inputs map through two downstream promoters. The gray shaded region contains the 5th to 95th percentiles of the distributions in (A). The purple line is the amplifying gene P_*micF*_; orange line is the filtering gene P_*inaA*_. **(D)** Schematic showing how MarA^+^ inputs map through two downstream promoters. **(E)** Channel capacity for the six downstream genes under wild type MarA levels. Heat map shows the channel capacity as a function of K_d_ and n. Experimentally-derived K_d_ and n values for the six genes are plotted. **(F)** Channel capacity for the six genes under MarA^+^ input levels.

While the previous results illustrate the differences in activation and transmitted noise, these findings need to be placed in the context of the biologically relevant levels of MarA. To investigate this we quantified the channel capacity of each promoter. Channel capacity is defined as the maximum mutual information—the potential of the input to inform the output [28]. The channel capacity reflects the ideal distribution of inputs through a target channel for maximizing mutual information [17]. To keep our calculations biologically grounded, we constrained the possible inputs by using estimated endogenous MarA levels. This is critical to our analysis because the physiologically relevant ranges of MarA are limited, and in some cases only span a narrow section of the downstream gene’s activation curve (Fig 1D and 1F). In order to quantify channel capacity for a given promoter, we divided our analysis into two cases that correspond to wild type and MarA^+^ levels. By mapping the distribution of GFP values that corresponds to wild type and MarA^+^ through our inducible system, we were able to estimate the MarA distribution that produced each downstream response (S4 Fig). Together, these two distributions represent physiologically relevant estimates of unstressed and stressed MarA levels (Fig 3A and 3B).

To provide intuition into the channel capacity of a promoter, we considered how MarA levels that correspond to wild type and MarA^+^ cells affect genes with amplifying versus filtering properties. At low levels of MarA, amplifying genes like P_*micF*_ transmit information well, proportionally mapping input to output. In contrast, the filtering genes like P_*inaA*_ map the same input to a narrow band of output (Fig 3C). The difference in width between the input and output distributions in the filtering gene corresponds to information loss. In contrast, under MarA^+^ conditions the channel capacity of the filtering gene P_*inaA*_ increases, while the amplifying gene P_*micF*_ has a lower channel capacity since the promoter saturates and high MarA values all map to the same output (Fig 3D).

We asked how the channel capacity varied for the six MarA-regulated genes under wild type and MarA^+^ conditions. We calculated the channel capacity as a function of K_d_ and n for the two genetic backgrounds (Fig 3E and 3F). Using parameters derived from experimental data, we plotted the location of each of the downstream genes on the channel capacity heat map. We found that for wild type MarA levels, downstream genes within the filtering class display lower channel capacity than those in the amplifying class (Fig 3E). As the input increases to MarA^+^ levels, we observed a shift and the filtering genes increased channel capacity, while amplifying genes decreased (Fig 3F).

Our calculations for channel capacity quantify what the maximum mutual information is for a bounded range of MarA inputs. Calculating the actual mutual information requires precise knowledge of the MarA input distributions. To estimate this, we calculated mutual information between the MarA distributions from the wild type and MarA^+^ strains and the downstream gene expression distributions produced by these inputs (S5 Fig). Despite the low channel capacity under wild type conditions for many downstream genes, the MarA input distribution is optimized to transmit information at near channel capacity for the filtering genes. However, these data also suggest that the wild type MarA distribution may not be taking full advantage of the amplifying promoters P_*micF*_ and P_*marRAB*_, though we note that the results are very sensitive to the input distributions (S6 Fig), which are produced here as estimates.

The amplifying or filtering characteristics of a promoter are determined by how MarA binds. Therefore, it is likely that altering the MarA binding sequence would have a profound effect on the activation profile of the promoter it regulates, and in turn, its transmitted noise and channel capacity. Moreover, although we observed two general classes of promoters, it may be possible to generate promoters with intermediate properties.

**Fig 4:**
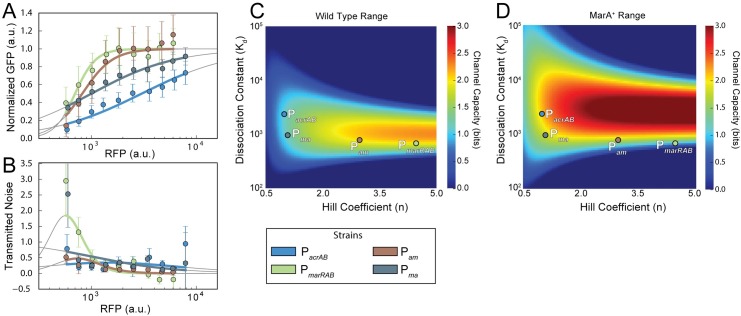
Chimeric promoters demonstrate intermediate activation, noise, and channel capacity values. **(A)** Chimeric promoter activation curves. Error bars show standard deviations of binned data. Solid lines are Hill function fits. P_*marRAB*_ and P_*acrAB*_ data are reproduced from Fig 2E. **(B)** Transmitted noise for chimeric promoters. Error bars show standard deviation from bootstrap resampling. **(C)** Channel capacity with wild type MarA levels. **(D)** Channel capacity with MarA^+^ input levels.

To investigate this, we created chimeric promoters by swapping the marbox sequences between P_*marRAB*_ and P_*acrAB*_. We constructed two chimeric promoters, which we denote P_*am*_ and P_*ma*_. In the P_*am*_ chimera we started with P_*acrAB*_ and replaced its marbox with that from P_*marRAB*_; in the P_*ma*_ chimera P_*marRAB*_ has the P_*acrAB*_ marbox. We quantified the activation and transmitted noise of these chimeric promoters as before (Fig 4A and 4B). We found that both chimeric promoters have K_d_ and n parameter values that fall between the two natural promoters, and the corresponding channel capacity is also intermediate as a result (Fig 4C and 4D). This shows that activation, and the transmitted noise and information properties that depend on it, are readily tunable through marbox mutations. This sequence could serve as an ideal target for evolutionary adaptation.

## Discussion

Genetic networks where one master regulator controls multiple downstream genes can efficiently respond to stress by customizing the response of individual genes based on their diverse functions. Using the multiple antibiotic resistance activator MarA as a case study, we show that its downstream targets are individually tailored in the way they respond to MarA and how they transmit noise and information.

Understanding the physiological context of MarA proved to be critical; for instance, we found downstream genes that amplified signals under only wild type levels of MarA (P_*micF*_ and P_*marRAB*_) and also examples that only show a response under high, non-physiological MarA conditions (P_*acrAB*_, P_*inaA*_, P_*sodA*_, and P_*tolC*_). These results argue that studies of stress response genes should be coupled with a concrete understanding of the appropriate cellular context. In our experiments with MarA we determined that there were two qualitative classes of downstream genes, which serve to increase variability or transmit critical signals. Ultimately, a cell’s ability to survive stress depends upon expression of multiple downstream genes in a coordinate fashion. The flexibility of MarA responses could balance multiple demands on a particular gene’s expression including cost, desired expression level, and noise. Further, the dissociation constant K_d_ and Hill coefficient n, are critical in setting a gene’s response. Our work with the chimeric promoters illustrates that these parameters are readily changed by altering the sequence of the marbox. We note that there are two MarA homologs, SoxS and Rob, that may play additional regulatory roles, further underscoring the need for context-dependent measurements [29].

While our experiments focused on activation, the underlying analysis can be extended to other classes of regulation. For instance, transmitted noise is proportional to the local slope of repressors just as it is in activators. As such, this study serves as a framework for contrasting how one gene controls many in the context of noise and information, unconstrained by the method of regulation. In the future it will also be interesting to examine the role of feedback, as previous research has shown that positive feedback has the potential to increase signal transmission without transmitting the associated noise [30].

Throughout our experiments, we show that each of the downstream genes behave differently in wild type and MarA^+^ strains. These two conditions correspond to approximations for stressed and unstressed states. Both P_*marRAB*_ and P_*micF*_ control regulatory molecules and therefore these amplifying genes may serve to increase diversity in unstressed conditions. As cells shift to stressed conditions, expression of these genes saturates. This could signify the transition from a bet-hedging state, where diversity is favored, to a state where all cells consistently express the target gene products. In contrast, the filtering genes only engage under high levels of MarA. Efflux pumps and other gene products controlled by these promoters are often costly to the cell and should only be expressed when needed [38].

We have demonstrated the flexibility of multi-gene regulation in stress response networks through a collection of single-cell experiments, computational simulations, and analytical analysis. Our results show how multiple downstream genes can display customized expression given the same input. Straightforward changes in promoter sequence can be used to change activation, noise, and information transmission properties, allowing for a diverse set of possible outcomes that can be tailored to optimize expression of specific gene products. Our findings on the plasticity and specificity of the MarA network provide insight into the role that master regulators can play in diverse stress response networks.

## Methods

### Strains and plasmids

All plasmids were derived from the BioBrick library described in [31]. In order to construct the downstream gene reporter plasmids (denoted P_*acrAB*_, P_*inaA*_, P_*marRAB*_, P_*micF*_, P_*sodA*_, and P_*tolC*_) we placed the promoter from each upstream of super-folder green fluorescent protein (*gfp*) (AddGene #63176). These transcriptional reporters were constructed on a plasmid containing the kanamycin resistance marker and low-copy SC101 origin of replication (from [31]).

For the activator/reporter experiments, we cotransformed the GFP reporter plasmid with a second plasmid containing the ampicillin resistance marker and medium-copy p15A origin of replication (pBbA5k from [31]). This plasmid places either *marA-rfp* or *rfp* under the control of the IPTG-inducible lacUV5 promoter. *marA-rfp* is a transcriptional fusion of *marA* and *rfp*.

We used three strains for experiments: wild type *E*. *coli* MG1655, and genetic variants Δ*marRAB* and MarA^+^. The Δ*marRAB* strain is described in [9]. In MarA^+^, we used transversion mutations that annihilate the MarR binding sites in the chromosomal copy of the *marRAB* promoter, preventing repression of the operon.

We constructed the chimeric reporter plasmids using P_*acrAB*_ and P_*marRAB*_ with marbox sequences from [7].

Further details on plasmids and strains is provided in Supplementary Information.

### Single cell fluorescence microscopy

Cultures were inoculated from single colonies and grown overnight at 37°C with 200 rpm shaking in LB medium with 30 μg/ml kanamycin (reporter-only) or 30 μg/ml kanamycin and 100 μg/ml carbenicillin (activator/reporter). Overnight cultures were diluted 1:100 in selective LB. For the activator/reporter experiments, we added 0, 10, 20, 30, 40, 50, 60, 70, 80, 90, 100, or 500 μM IPTG and grew cultures for four hours (Supplementary Information and S1 Fig). For the reporter-only experiments, we grew cultures for two hours before adding either salicylate (1 or 3 mM) or ciprofloxacin (2 or 4 μg/L), then grew them for an additional two hours. Wild type and MarA^+^ reporter-only strains were grown for four hours without the addition of inducers.

For microscopy images, we placed cells on 1.5% MGC low melting temperature agarose pads [32]. We used a Nikon Instruments Ti-E microscope to image the cells at 100× magnification. Three images were taken of each pad to ensure that at least 100 cells were imaged under each growth condition. Custom MATLAB scripts were used to extract fluorescence data from individual cells. For the activator/reporter experiments, fluorescence values for cells from all IPTG levels were combined and then binned according to their RFP levels (S1 Fig).

### Activation

To quantify activation of each downstream gene, we used Hill functions:

$$B=\frac{\alpha }{\beta }\left(\frac{{\left(\frac{A}{K_{d}}\right)}^{n}}{1+{\left(\frac{A}{K_{d}}\right)}^{n}}\right)+\frac{c}{\beta }$$ (1)

where A is MarA and B is the downstream gene product. α is the promoter strength, K_d_ is the dissociation constant, n is the Hill coefficient, c is the basal expression level, and β is the degradation and dilution rate. The functions for calculating the normalized level of downstream gene product (used in Fig 2) subtract the basal expression and normalize (Supplementary Information).

### Transmitted noise

We calculated transmitted noise using two independent methods in this study. First, transmitted noise is calculated from experimental data as the ratio of the noise in the downstream gene output (B) over the noise in the MarA input (A) [30]. We note that this quantity is sometimes referred to as ‘noise amplification’.

$$\eta_{transmitted}=\frac{\left(\frac{\sigma_{B}}{\mu_{B}}\right)}{\left(\frac{\sigma_{A}}{\mu_{A}}\right)}-S$$ (2)

Noise in an individual gene is calculated as the coefficient of variation, which is the standard deviation (σ) divided by the mean (μ) for a given gene product level [30,33]. However, we needed to account for noise sources not coming directly from fluctuations in MarA, such as those from intrinsic and extrinsic noise [34]. The term S is equal to the noise of the downstream gene without regulation by MarA, and is necessary to fit the analytical solution to the experimentally measured transmitted noise. S does not vary as a function of MarA and is the sum of intrinsic and extrinsic noise sources that are independent of upstream gene regulation. Statistics were determined by bootstrap resampling of one third of the population 100 times.

Analytically, transmitted noise is equal to the local slope of the activation curve, normalized by the values of the function about that point [19]. Mathematically, this is equal to the slope of the logarithmic transform of the Hill function [30,35].

$$\eta_{transmitted}=\frac{dB}{dA}*\frac{\mu_{A}}{\mu_{B}}=\frac{d(lnB)}{d(lnA)}$$ (3)

We used this function to calculate the analytic solutions in all noise plots.

### Fitting parameters for transmitted noise and Hill functions

Because the transmitted noise is equal to the local slope of the activation curve, Hill functions and transmitted noise curves share the same parameters [19]. We simultaneously fit both curves to experimental data using a differential evolution algorithm with a custom fitness function [36]. For fitness function and exact values of fitted parameters, see Supplementary Information.

### Stochastic simulation

We modeled the input A and the downstream products B by:

$$\dot{A}=\alpha_{A}-\beta A+I_{A}$$ (4)

$$\dot{B}=\alpha \frac{{\left(\frac{A}{K_{d}}\right)}^{n}}{1+{\left(\frac{A}{K_{d}}\right)}^{n}}-\beta B$$ (5)

where α is the promoter strength, K_d_ is the dissociation constant, and n is the Hill coefficient. In addition, α_A_ is the production rate of A and β describes protein degradation and dilution for both A and B. We simulated intrinsic noise of the input protein I_A_ using an Ornstein-Uhlenbeck process [37]. The intrinsic noise of the input has a standard deviation of $\sqrt{\alpha }$. The correlation time of this noise calculated as T_int_/ln(2), with T_int_ of 5 minutes [32]. As with the experimental data, the level of input protein (α_A_) was varied through a range of possible inputs (30 log spaced values). The parameters used and details of the stochastic simulation are given in Supplementary Information.

### Channel capacity

The channel capacity (I^*^) is dependent on the relationship between input and output and is calculated using the functions from [17,19,20,28]:

$$I^{*}(A;B)={log}_{2}(Z)+X$$ (6)

$$Z=\int_{A_{min}}^{A_{max}}{\left[\frac{{\left(dB/dA\right)}^{2}}{B+A_{0}*A*{\left(dB/dA\right)}^{2}}\right]}^{\frac{1}{2}}dA$$ (7)

X is a constant that is independent of the parameters of the downstream promoters. It is introduced by the small noise approximation implicit in this calculation of channel capacity. A_min_ and A_max_ describe the minimum and maximum input values, which we determine from experimental data by mapping the output from wild type and MarA^+^ to the data from the activator/report experiments (S4 Fig) using the 5th to 95th percentiles from these distributions. A_0_ is a scaling term for the concentration of the activator to match experimental results. For further details see Supplementary Information.

## Supporting Information

S1 Text Supplementary materials and methods including strains, plasmids, primers, data parsing methods, function fitting methodology, supplementary equations, and simulation parameters. (PDF)

S1 Fig P_*micF*_ response to MarA activation via IPTG induction. Fluorescence data from single cells from three microscopy images at each concentration of IPTG were combined and then logarithmically binned along the x-axis. Gray bars indicate bin demarcations. For each of these bins, we calculated mean and standard deviation for all cells contained within and these values are shown in black, where the error bars are standard deviation. The points shown in black were only calculated for bins with greater than 25 cells.
(TIF)

S2 Fig P_*micF*_ reporter with *marA-rfp* and *rfp* control. Multiple snapshots were combined to estimate the response of both strains to varying MarA levels as described in S1 Fig. The black dots represent mean and standard deviation of cells within each bin, while the black lines represent best fit Hill functions. The gray lines are these functions extrapolated beyond the experimental range. The control demonstrates a near zero slope, suggesting that without the *marA* gene, IPTG induction does not elicit a response from P_*micF*_.
(TIF)

S3 Fig Computational and analytical results for downstream promoter response as a function of α/β. **(A)** The activation response of five different promoters with varying α/β values, in addition to a control gene that is unregulated by the input. Dots show the mean and standard deviation generated using a stochastic simulation, while the colored lines are the analytic solutions for activation over the given input range. The gray lines are the analytical solutions outside of the stochastic simulation range. **(B)** Transmitted noise for the downstream genes. The dots are transmitted noise calculated as the coefficient of variation of B over the coefficient of variation of A. Error bars for the estimates were determined by bootstrapping. Because the only source of noise in this simulation is transmitted noise, S = 0. The lines show the analytical solution to the noise function given by the local slope of the activation curve.
(TIF)

S4 Fig Estimating MarA levels in wild type and MarA^+^ conditions. **(A)** Fluorescence distributions for reporter-only experiments with P_*micF*_ in both wild type and MarA^+^ strains. **(B)** Activator/reporter data for P_*micF*_ is shown in purple. For all cells in each of the distributions in (A), we found the cell in the P_*micF*_ activator/reporter data with the closest level of RFP. We refer to these as the “nearest neighbors” from the activator/repressor data set and the corresponding cells for the wild type and MarA^+^ distributions are shown in pink and teal. **(C)** Using the nearest neighbors, we collected the RFP values from these cells and generated the corresponding probability distributions. **(D-E)** We repeated the process above for all downstream genes given (D) wild type and (E) MarA^+^ inputs. P_*marRAB*_ samples were not included in these estimations as MarA^+^ also over-expresses MarR, a repressor of *marRAB*, preventing it from accurately reporting MarA levels. **(F)** We summed the values for all downstream reporters to estimate the underlying MarA levels in the wild type and MarA^+^ strains.
(TIF)

S5 Fig Estimated mutual information between MarA and downstream genes. Grey bars represent the channel capacity of the downstream promoters (values from Fig 3E and 3F). Colored bars show mutual information between estimated MarA input distributions and downstream targets (input values estimated as shown in S4D and S4E Fig). **(A)** Estimated wild type levels and **(B)** estimated MarA^+^ levels of MarA.
(TIF)

S6 Fig Mutual information vs. bounded channel capacity. **(A)** Transfer function of P_*micF*_ with grey bar indicating bounded region for channel capacity. **(B)** Three examples of input distributions. **(C)** Input distributions mapped through transfer function shown in (A). **(D)** Mutual information as a fraction of channel capacity for each of the input distributions. Grey bars are the bounded channel capacity, which are the same for all, while the colored bars are the calculated mutual information for each of the input distributions and their mapped outputs.
(TIF)
