# Supplementary material for: Customized Regulation of Diverse Stress Response Genes by the Multiple Antibiotic Resistance Activator MarA
Source: PLoS Comput Biol. 2017 Jan 6;13(1):e1005310. doi: 10.1371/journal.pcbi.1005310 (PMC5257004; doi:10.1371/journal.pcbi.1005310)
Supplement: S1 Text — (PDF) [file pcbi.1005310.s001.pdf]

# Supplementary Information

Customized Regulation of Diverse Stress Response Genes by the Multiple Antibiotic Resistance Activator MarA

N. A. Rossi, M. J. Dunlop

# 1 Strains and Plasmids

## 1.1 Strains

The MarA$^+$ strain is derived from *Escherichia coli* MG1655, with modifications to overexpress MarA. The chromosomal copies of the two MarR binding sites in the *marRAB* promoter are inactivated, removing negative feedback by MarR. This inactivation was accomplished by placing transversion mutations at these sites. The mutated sequences were derived from [1], where they are listed as "TV -14 to -18" and "TV +11 to 15." The new *marRAB* promoter is:
GCATCGCATTGAACAAAACTTGAACCGATTTAGCAAAACGTGGCATCGGTCAATTCA
TTCATTTGACTTATACTTGCCTG**TTACC**TATTATCCCCTGCAACTAATTAC**GGTAA**AGGG
C AACTAAT GTGAAAAGTACCAGCGATCTGTTCAATGAAATTATT
where bold letters indicate the transversion mutations, while underlining indicates MarR binding sites. We used homologous recombination to insert this modified promoter following [2], then cured the resistance marker.

## 1.2 Plasmids

Downstream Gene Reporter Plasmids

All plasmids were constructed using Gibson assembly [3]. For the transcriptional reporters, we amplified the promoters of the downstream genes and included all known binding sites. The primers typically terminated at the transcriptional start site and were joined to a synthetic ribosome binding site and the *gfp* gene. The only exception to this is $P_{marRAB}$, which extends beyond the transcriptional start site of the promoter in order to include the second MarR binding site. The ribosome binding site and plasmid backbone were taken from pBbSvk-cfplaa described in [4]. All primers used to amplify promoters are listed in Table S1.

*marA-rfp* and *rfp* Control

The *rfp* control used in Fig. S1 uses the plasmid pBbA5a-*rfp* from [5]. For the activator/reporter experiments this plasmid was modified to produce pBbA5a-*marA-rfp*. To construct this, the *marA* gene and an additional ribosome binding site were inserted directly upstream of *rfp*.

Chimeric Reporters

We constructed the chimeric reporters using site-directed mutagenesis to modify the MarA binding sites from plasmids for $P_{acrAB}$ and $P_{marRAB}$. The primers used for the mutagenesis are listed in Table S2. Underlined text shows the *acrAB* and *marRAB* marboxes inserted during site-directed mutagenesis. Bold text corresponds to the sequence where the primers bind to the original tem-

plate. $P_{am}$ is the $P_{acrAB}$ promoter with the *marRAB* marbox; $P_{ma}$ is the $P_{marRAB}$ promoter with the *acrAB* marbox.

| Construct | Primers |
|---|---|
| $P_{acrAB}$ | GCCAGTAGATTGCACCGCG<br>TCGTGCTATGGTACATACATTCACA |
| $P_{micF}$ | GACAGTACTTTAACTTTCATGTTATTAAC<br>ATGACGGTAATAAATAAAGTTAATGA |
| $P_{inaA}$ | CAATGCTTTTCAGCGTTAACTCTG<br>ACGACAATGACTATAGGTGGT |
| $P_{marRAB}$ | GGTTGTTATCCTGTGTATCTGG<br>ATTAGTTGCCCTGGCAAG |
| $P_{sodA}$ | GGCAATCACGGCATTAAG<br>TTGGTTCATTATAGTTAATTAAATG |
| $P_{tolC}$ | CGTTTGACTGCCGTTTG<br>TGCGGATTCTAGCAGAAGC |

Table S1: Primers for Construction of Transcriptional Reporter Plasmids

| Construct | Primers |
|---|---|
| $P_{ma}$ | ATGGCACGAAAAACCAAACA**CGGTCAATTCATTCATTTGAC**<br>**GGTTCAAGTTTTGTTCAATGC** |
| $P_{am}$ | GATTTAGCAAAACGTGGCAT**ATGTTCGTGAATTTACAGGC**<br>**AGAAGCGCAAGAAACGC** |

Table S2: Primers for Construction of Chimeric Reporter Plasmids

# 2 Combining and Binning Values

To characterize each promoter we induced *marA-rfp* expression under a range of IPTG concentrations, combined the data, and binned the values to generate output statistics based on input values

(Fig. S1) . Each data point was calculated by binning the IPTG-induced activator/reporter data logarithmically, then computing statistics (mean and standard deviation, $\mu$ and $\sigma$) on the data within that bin.

# 3 Activation

## 3.1 Activation Function

In order to model how a downstream gene responds to activation by MarA, we used

$$\frac{dB}{dt} = \alpha\left(\frac{(\frac{A}{K_d})^n}{1+(\frac{A}{K_d})^n}\right) + c - \beta B, \tag{1}$$

where the steady state solution is

$$B = \frac{\alpha}{\beta}\left(\frac{(\frac{A}{K_d})^n}{1+(\frac{A}{K_d})^n}\right) + \frac{c}{\beta}. \tag{2}$$

Here, $A$ is the MarA concentration and $B$ is the downstream gene product concentration. $\alpha$ is the promoter strength, $\beta$ is the dilution/degradation rate, $K_d$ is the dissociation constant of MarA with the downstream gene, $n$ is the Hill coefficient, and $c$ is the basal level of expression.

When plotting the activation in Figs. 2 and 4 of the main text, we use a normalized version of this function with the basal level subtracted:

$$B_{norm} = \frac{(\frac{A}{K_d})^n}{1+(\frac{A}{K_d})^n}. \tag{3}$$

This function depends on the promoter specific parameters, not on the behavior of the specific gene product regulated by the promoter.

## 3.2 Control for Cross-talk in Activation Measurements

In order to verify that there was no cross-talk between our RFP and GFP channels, we constructed a control strain bearing an IPTG inducible *rfp* gene without *marA*. Regardless of the level of RFP, we observed no increase in GFP expression (Fig. S2).

# 4 Fitting Parameters

In order to simultaneously fit both the activation and transmitted noise curves, we built a fitness function and used the differential evolution algorithm to fit parameter values [6]. This function performs an element-wise comparison between the experimentally calculated values (input and output; A and B) and the analytical solutions for the fitted functions for each bin. The experimental values were calculated using the procedure shown in Fig. S1. The analytic solutions were

| Reporter | $\alpha$ | $K_d$ | n | c | S |
|---|---|---|---|---|---|
| $P_{acrAB}$ | 2051 | 2828 | 1.00 | 614 | 1.17 |
| $P_{micF}$ | 8785 | 822 | 4.10 | 500 | 2.27 |
| $P_{inaA}$ | 5676 | 9832 | 1.00 | 566 | 1.17 |
| $P_{marRAB}$ | 7895 | 700 | 4.47 | 1796 | 1.97 |
| $P_{sodA}$ | 2840 | 4475 | 1.12 | 708 | 1.59 |
| $P_{tolC}$ | 3445 | 9994 | 1.50 | 884 | 0.90 |
| $P_{am}$ | 783 | 822 | 3.00 | 902 | 1.23 |
| $P_{ma}$ | 6553 | 1000 | 1.15 | 45 | 1.41 |

Table S3: Parameters Fitted to Experimental Data

calculated using the output and noise equations (Eqns. 1 and 3 in the main text). $\mu_{A_i}$ is the mean of the input (RFP channel) for the $i^{th}$ bin.

$$cost = \sum_{i=1}^{N_{bins}} \left( \left( \frac{B - \mu_{B_i}}{\mu_{B_i}} \right)^2 + \left( \frac{\left(\frac{dB}{dA} * \frac{A}{B} + S\right) - \frac{\mu_{B_i}/\sigma_{B_i}}{\mu_{A_i}/\sigma_{A_i}}}{\frac{\mu_{B_i}/\sigma_{B_i}}{\mu_{A_i}/\sigma_{A_i}}} \right)^2 \right) \tag{4}$$

Using this cost function, we fit parameter values for each of our reporter strains (Table S3). Note that for the sake of simplicity, $\beta$ was not fit as a free parameter since it simply acts as a scaling term. Instead we set $\beta = 1$ and fit $\alpha$ and $c$. While the experimental data only occupies a portion of the entire Hill curve, our fitting function fits both the activation and noise simultaneously. This allows for a good fit without explicitly assuming a maximum expression level for any of the downstream genes or constraining the parameters in any other way. Note as well that the values of $K_d$ here are in units of arbitrary fluorescence - which correspond to the concentration of MarA. The exact values are a function of the specific experimental protocol, but the relative values between each downstream gene will be maintained regardless of method.

# 5 Noise in Mapped MarA Distributions

The coefficient of variation of the estimated wild type MarA distribution was 0.48 versus 0.65 in the MarA+ strain. These are on the high-end of gene expression coefficients of variation in *E. coli*, which usually range from 0.2 to 0.5 [7]. Transmitted noise through each of the downstream genes functions as a multiplication of this value.

# 6 Computational Modeling

We modeled noisy expression of a transcription factor $A$ using

$$\dot{I}_A = -\kappa I_A + \lambda\eta \tag{5}$$

where $I_A$ is the intrinsic noise of $A$. This is modeled using an Ornstein-Uhlenbeck processes [8]. Here, $\kappa$ defines the time scale of the noise and scales with correlation time and $\lambda$ depends on the standard deviation of the noise [8]. $\eta$ is a zero mean white noise random variable.

| Parameter | Value | Notes |
|---|---|---|
| $\alpha_A$ | 30 logarithmically-spaced values between $10^2$ and $10^5$ | input promoter strength |
| $\kappa$ | 0.933 | noise time scale |
| $\lambda$ | $\sqrt{\alpha_A * (1-\kappa^2)}$ | promoter strength noise |
| $\alpha$ | 1, unless noted otherwise | downstream promoter strength |
| $\beta$ | 1 | dilution/degradation |
| $c$ | 1 | basal expression rate |
| $K_d$ | $10^3$, unless noted otherwise | dissociation constant of activator and downstream gene |
| n | 2, unless noted otherwise | Hill coefficient of downstream gene |

Table S4: Parameters used in Computational Simulation

Initial conditions were set at the steady state solutions for $A$ and $B$ given by

$$A_{ss} = \frac{\alpha_A}{\beta} \quad (6)$$

$$B_{ss} = \frac{\alpha}{\beta}\frac{(\frac{A}{K_d})^n}{1+(\frac{A}{K_d})^n} + \frac{c}{\beta}. \quad (7)$$

Simulation were run for 10,000 minutes of simulation time and values were then used to create distributions for each protein species. The differential equations were integrated using Euler's method with a time-step of 0.5 minutes.

Either varying $\alpha$ or $\beta$ has the capacity to affect activation and noise transmission as shown in Fig. S3.

# 7 Information

## 7.1 Physiologically Relevant Ranges of MarA

The computation of channel capacity for each downstream gene uses the physiological ranges of MarA from the wild type and MarA$^+$ strains. However, we do not directly measure MarA levels, but rather the downstream response. To estimate endogenous MarA levels, we mapped the results from the reporter-only experiments in wild type and MarA$^+$ strains through our activator/reporter data to obtain estimates of the wild type and MarA$^+$ levels. An example of this is shown in Fig. S4. For each cell's fluorescence level in the reporter-only experiments, we matched the nearest neighbors for wild type and MarA$^+$ conditions in the activator/reporter experiment. The

RFP values from these points served as estimates for the MarA level producing that downstream response. By repeating this process for each downstream gene, we generated independent estimates for MarA levels in wild type and MarA$^+$ conditions. As the MarA levels are the same in each of the wild type and MarA$^+$ experiments, we combined data to estimate the underlying MarA distributions.

## 7.2 Channel Capacity

We calculated channel capacity using

$$I^*(A;B) = log_2(Z) + X \tag{8}$$

$$Z = \int_{A_{min}}^{A_{max}} \left| \frac{(dB/dA)^2}{B + A_0 * A * (dB/dA)^2} \right|^{\frac{1}{2}} dA, \tag{9}$$

where $I^*(A;B)$ is the channel capacity given input $A$ (MarA) and output $B$ (downstream gene). In our calculations we used the normalized values of B from Eqn. 3. $A_0$ is the natural scale of concentration of the activator molecule, which we have set to 1000 for it to scale with the order of magnitude of our experimental measurements taken as arbitrary units of fluorescence. The constant $X$ is based on the expansion from [9]:

$$X = log_2 \left( \left( \frac{N_{max}}{2\pi e} \right)^{\frac{1}{2}} \right), \tag{10}$$

where $N_{max}$ is the maximum number of independent molecules produced from a downstream promoter. The number of independent molecules produced from a promoter is equal to the natural scale of concentration and agrees with the order of magnitude of average protein copy number in *E. coli* [10].

## 7.3 Mutual Information

Mutual information for Fig. S5 and Fig. S6 is calculated using the following equation from [11]

$$I(A;B) = \int P(A)dA \int P(B|A) log_2 (\frac{P(B|A)}{P(B)}) dB. \tag{11}$$

Where $P(A)$ and $P(B)$ are the input and output probability distributions, respectively. In order to calculate this value from our experimental data we binned the data following the methods from [12] and calculated it as discrete mutual information. The number of bins scaled with the number of samples following the equation below from [12]

$$N_{bins} = \sqrt{\frac{N_{samples}}{5}}. \tag{12}$$

# References


[1] Robert G Martin and Judah L Rosner. Transcriptional and translational regulation of the marrab multiple antibiotic resistance operon in escherichia coli. *Molecular microbiology*, 53(1):183–191, 2004.

[2] Kirill A Datsenko and Barry L Wanner. One-step inactivation of chromosomal genes in escherichia coli k-12 using pcr products. *Proceedings of the National Academy of Sciences*, 97(12):6640–6645, 2000.

[3] Daniel G Gibson, Lei Young, Ray-Yuan Chuang, J Craig Venter, Clyde A Hutchison, and Hamilton O Smith. Enzymatic assembly of dna molecules up to several hundred kilobases. *Nature methods*, 6(5):343–345, 2009.

[4] Imane El Meouche, Yik Siu, and Mary J Dunlop. Stochastic expression of a multiple antibiotic resistance activator confers transient resistance in single cells. *Scientific reports*, 6:19538, 2016.

[5] Taek Soon Lee, Rachel A Krupa, Fuzhong Zhang, Meghdad Hajimorad, William J Holtz, Nilu Prasad, Sung Kuk Lee, and Jay D Keasling. Bglbrick vectors and datasheets: a synthetic biology platform for gene expression. *Journal of biological engineering*, 5(1):1, 2011.

[6] Rainer Storn and Kenneth Price. Differential evolution–a simple and efficient heuristic for global optimization over continuous spaces. *Journal of global optimization*, 11(4):341–359, 1997.

[7] Olin K. Silander, Nela Nikolic, Alon Zaslaver, Anat Bren, Ilya Kikoin, Uri Alon, and Martin Ackermann. A genome-wide analysis of promoter-mediated phenotypic noise in escherichia coli. *PLOS Genetics*, 8(1):1–13, 01 2012.

[8] Daniel T Gillespie. Exact numerical simulation of the ornstein-uhlenbeck process and its integral. *Physical review E*, 54(2):2084, 1996.

[9] Gašper Tkačik, Aleksandra M Walczak, and William Bialek. Optimizing information flow in small genetic networks. *Physical Review E*, 80(3):031920, 2009.

[10] Mary Ann Moran, Brandon Satinsky, Scott M Gifford, Haiwei Luo, Adam Rivers, Leong-Keat Chan, Jun Meng, Bryndan P Durham, Chen Shen, Vanessa A Varaljay, et al. Sizing up metatranscriptomics. *The ISME journal*, 7(2):237–243, 2013.

[11] Gašper Tkačik, Curtis G Callan, and William Bialek. Information flow and optimization in transcriptional regulation. *Proceedings of the National Academy of Sciences*, 105(34):12265–12270, 2008.

[12] Christopher J Cellucci, Alfonso M Albano, and Paul E Rapp. Statistical validation of mutual information calculations: Comparison of alternative numerical algorithms. *Physical Review E*, 71(6):066208, 2005.
