# Supplementary figures and images for: Customized Regulation of Diverse Stress Response Genes by the Multiple Antibiotic Resistance Activator MarA
Source: PLoS Comput Biol. 2017 Jan 6;13(1):e1005310. doi: 10.1371/journal.pcbi.1005310 (PMC5257004; doi:10.1371/journal.pcbi.1005310)

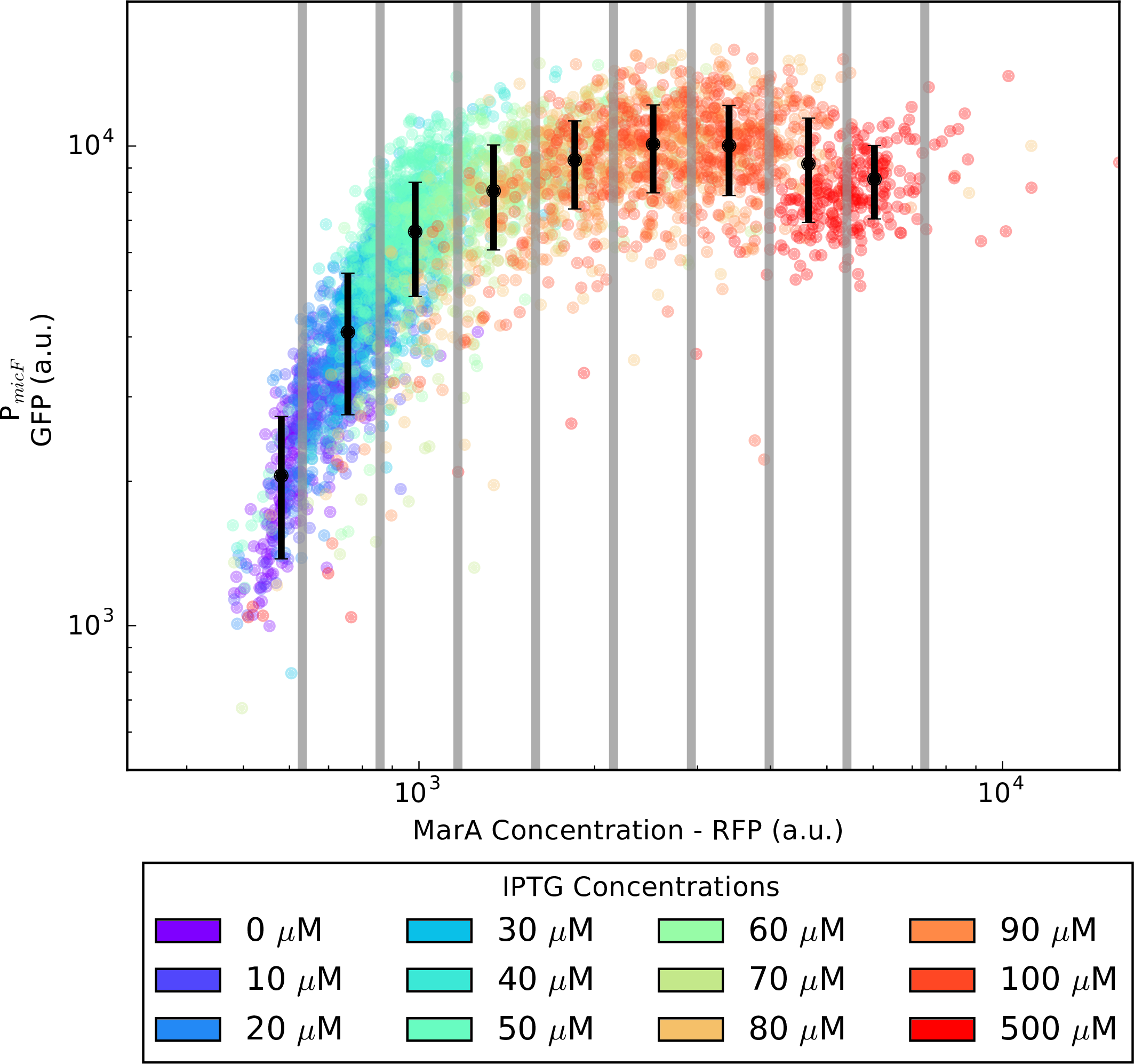

Supplement: S1 Fig — Fluorescence data from single cells from three microscopy images at each concentration of IPTG were combined and then logarithmically binned along the x-axis. Gray bars indicate bin demarcations. For each of these bins, we calculated mean and standard deviation for all cells contained within and these values are shown in black, where the error bars are standard deviation. The points shown in black were only calculated for bins with greater than 25 cells. (TIF) [file pcbi.1005310.s002.tif]

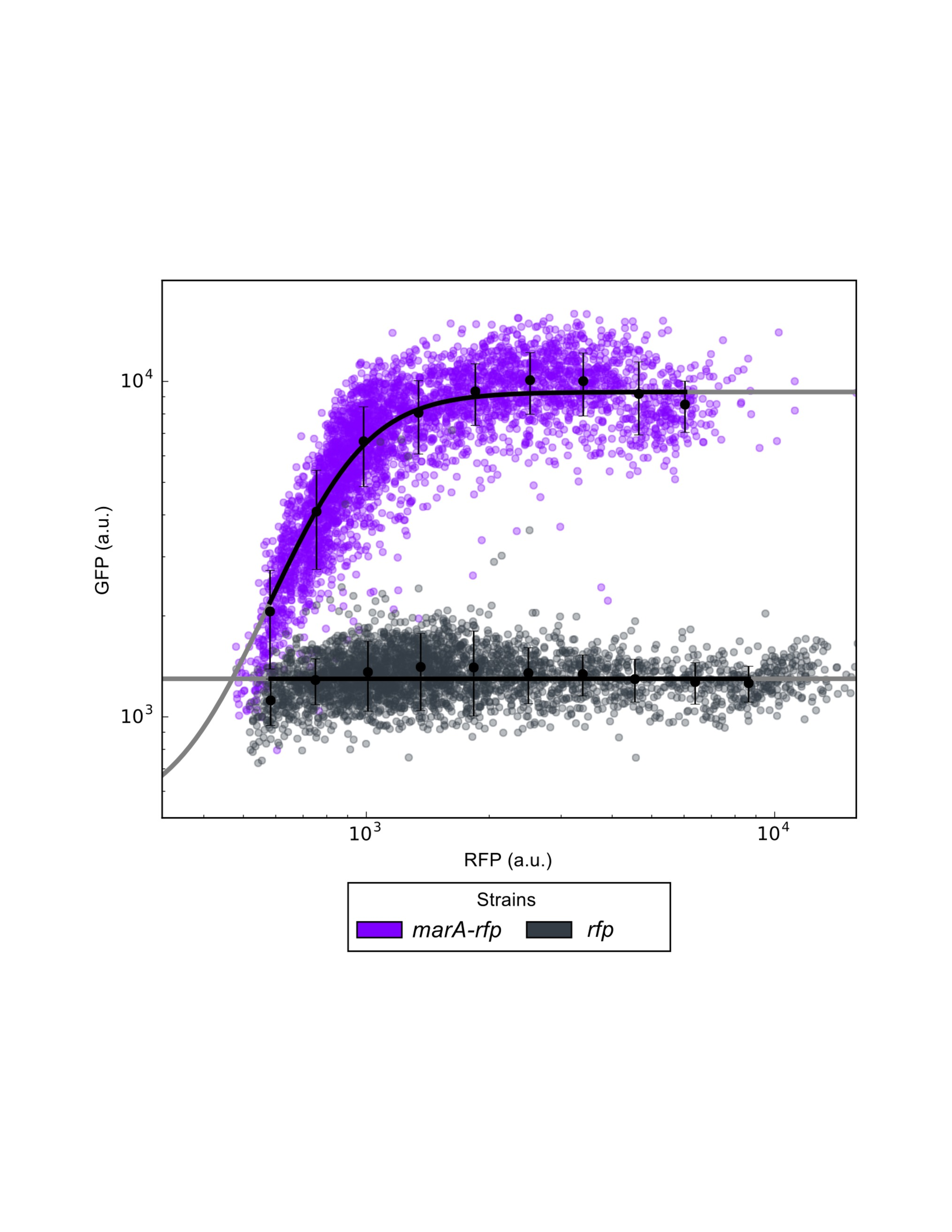

Supplement: S2 Fig — Multiple snapshots were combined to estimate the response of both strains to varying MarA levels as described in S1 Fig. The black dots represent mean and standard deviation of cells within each bin, while the black lines represent best fit Hill functions. The gray lines are these functions extrapolated beyond the experimental range. The control demonstrates a near zero slope, suggesting that without the marA gene, IPTG induction does not elicit a response from PmicF. (TIF) [file pcbi.1005310.s003.tif]

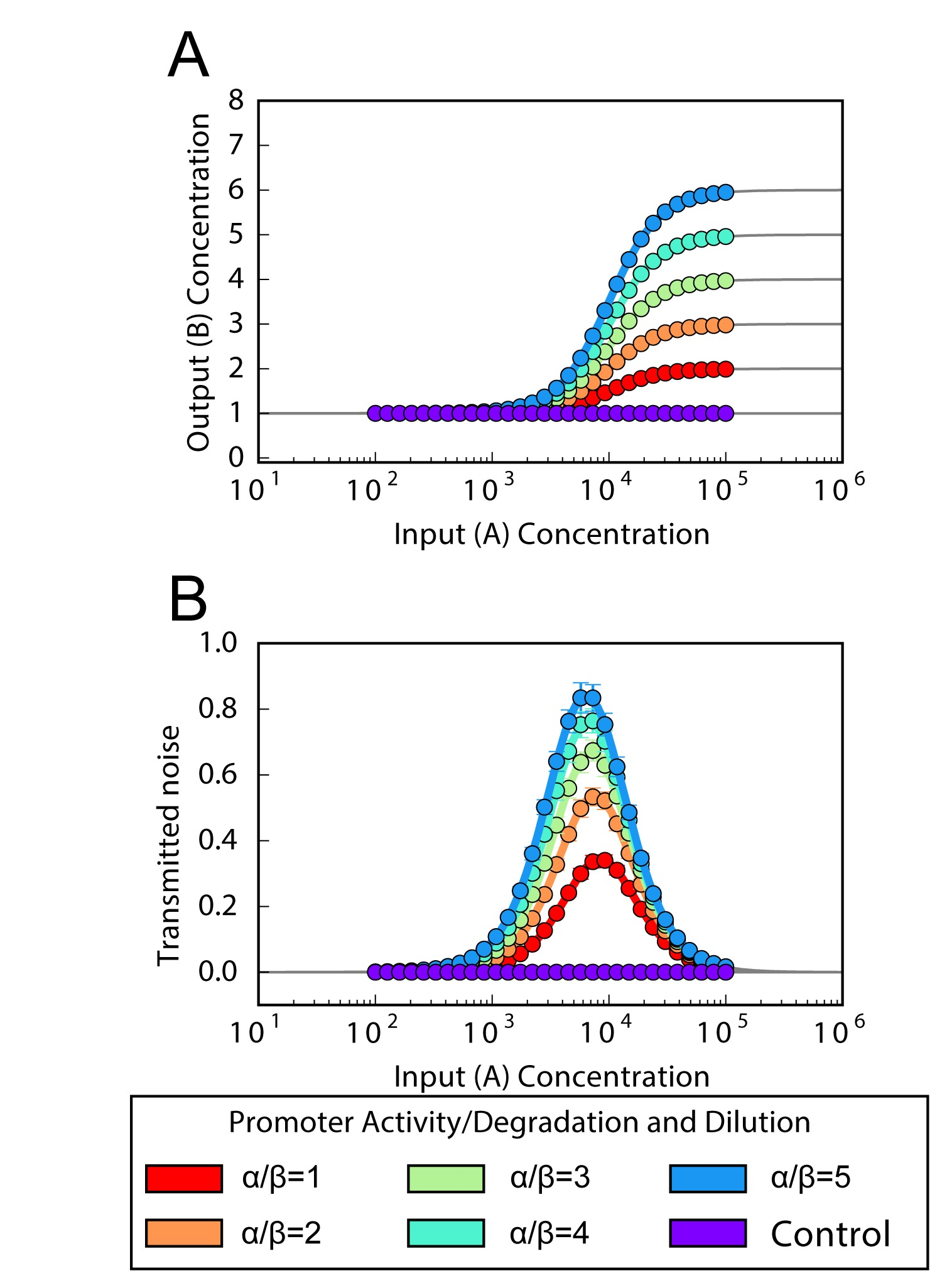

Supplement: S3 Fig — (A) The activation response of five different promoters with varying α/β values, in addition to a control gene that is unregulated by the input. Dots show the mean and standard deviation generated using a stochastic simulation, while the colored lines are the analytic solutions for activation over the given input range. The gray lines are the analytical solutions outside of the stochastic simulation range. (B) Transmitted noise for the downstream genes. The dots are transmitted noise calculated as the coefficient of variation of B over the coefficient of variation of A. Error bars for the estimates were determined by bootstrapping. Because the only source of noise in this simulation is transmitted noise, S = 0. The lines show the analytical solution to the noise function given by the local slope of the activation curve. (TIF) [file pcbi.1005310.s004.tif]

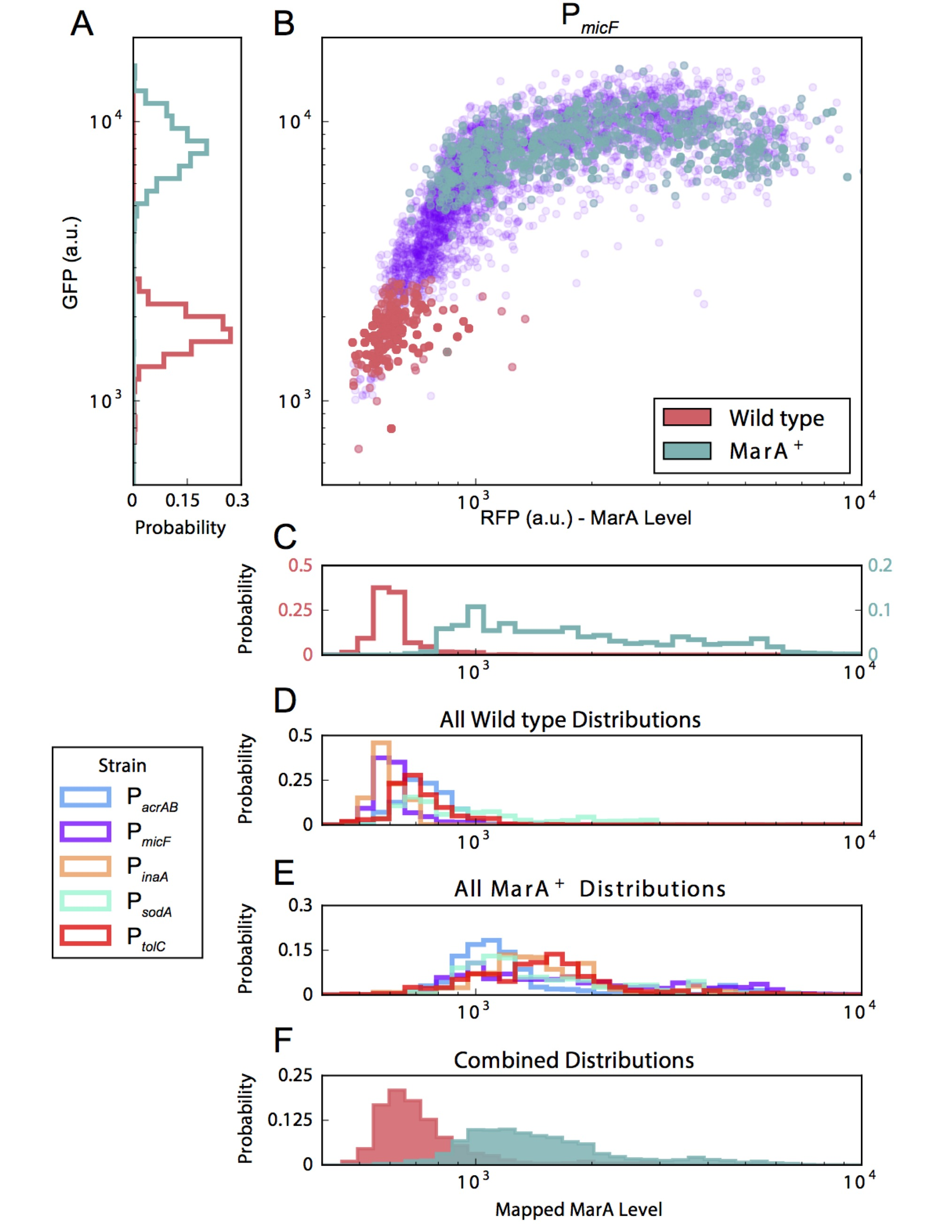

Supplement: S4 Fig — (A) Fluorescence distributions for reporter-only experiments with PmicF in both wild type and MarA+ strains. (B) Activator/reporter data for PmicF is shown in purple. For all cells in each of the distributions in (A), we found the cell in the PmicF activator/reporter data with the closest level of RFP. We refer to these as the “nearest neighbors” from the activator/repressor data set and the corresponding cells for the wild type and MarA+ distributions are shown in pink and teal. (C) Using the nearest neighbors, we collected the RFP values from these cells and generated the corresponding probability distributions. (D-E) We repeated the process above for all downstream genes given (D) wild type and (E) MarA+ inputs. PmarRAB samples were not included in these estimations as MarA+ also over-expresses MarR, a repressor of marRAB, preventing it from accurately reporting MarA levels. (F) We summed the values for all downstream reporters to estimate the underlying MarA levels in the wild type and MarA+ strains. (TIF) [file pcbi.1005310.s005.tif]

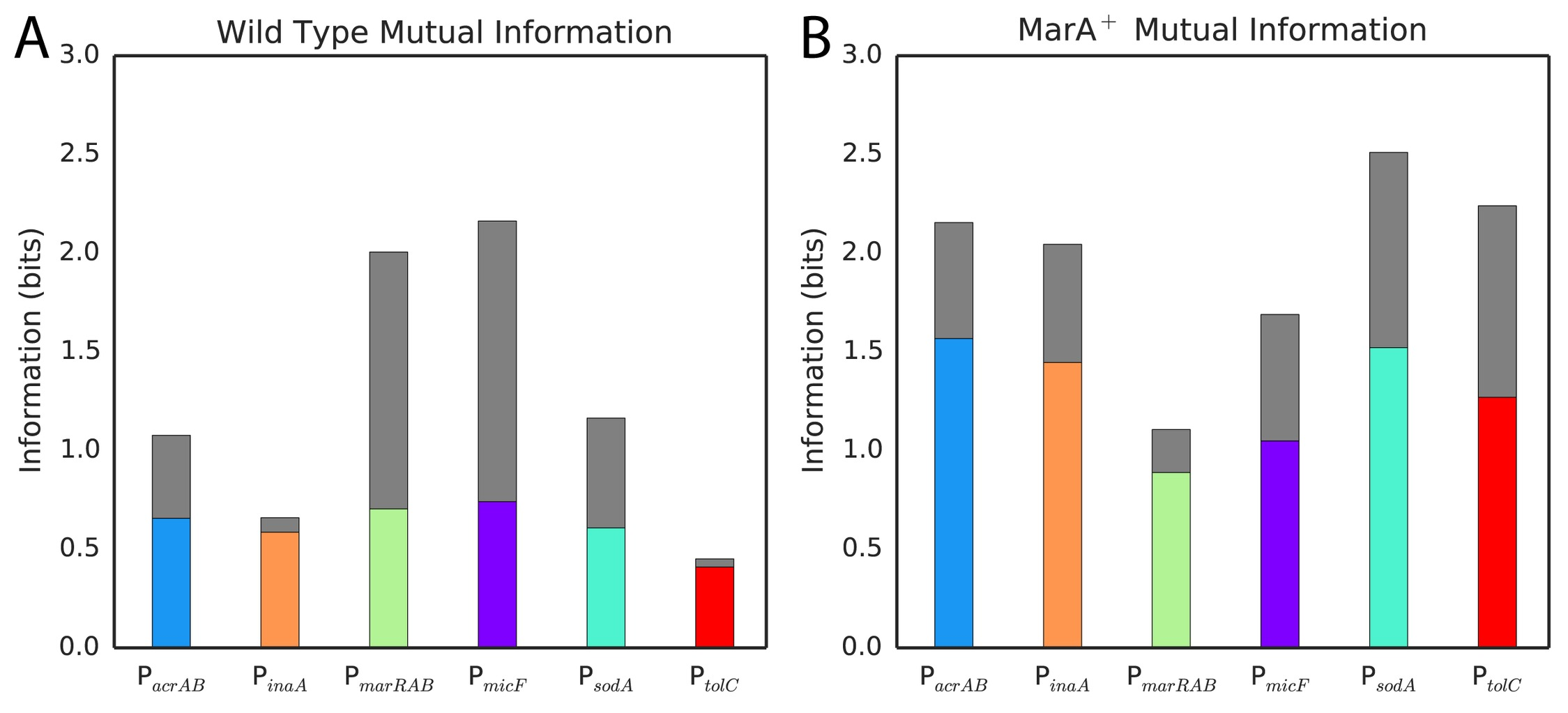

Supplement: S5 Fig — Grey bars represent the channel capacity of the downstream promoters (values from Fig 3E and 3F). Colored bars show mutual information between estimated MarA input distributions and downstream targets (input values estimated as shown in S4D and S4E Fig). (A) Estimated wild type levels and (B) estimated MarA+ levels of MarA. (TIF) [file pcbi.1005310.s006.tif]

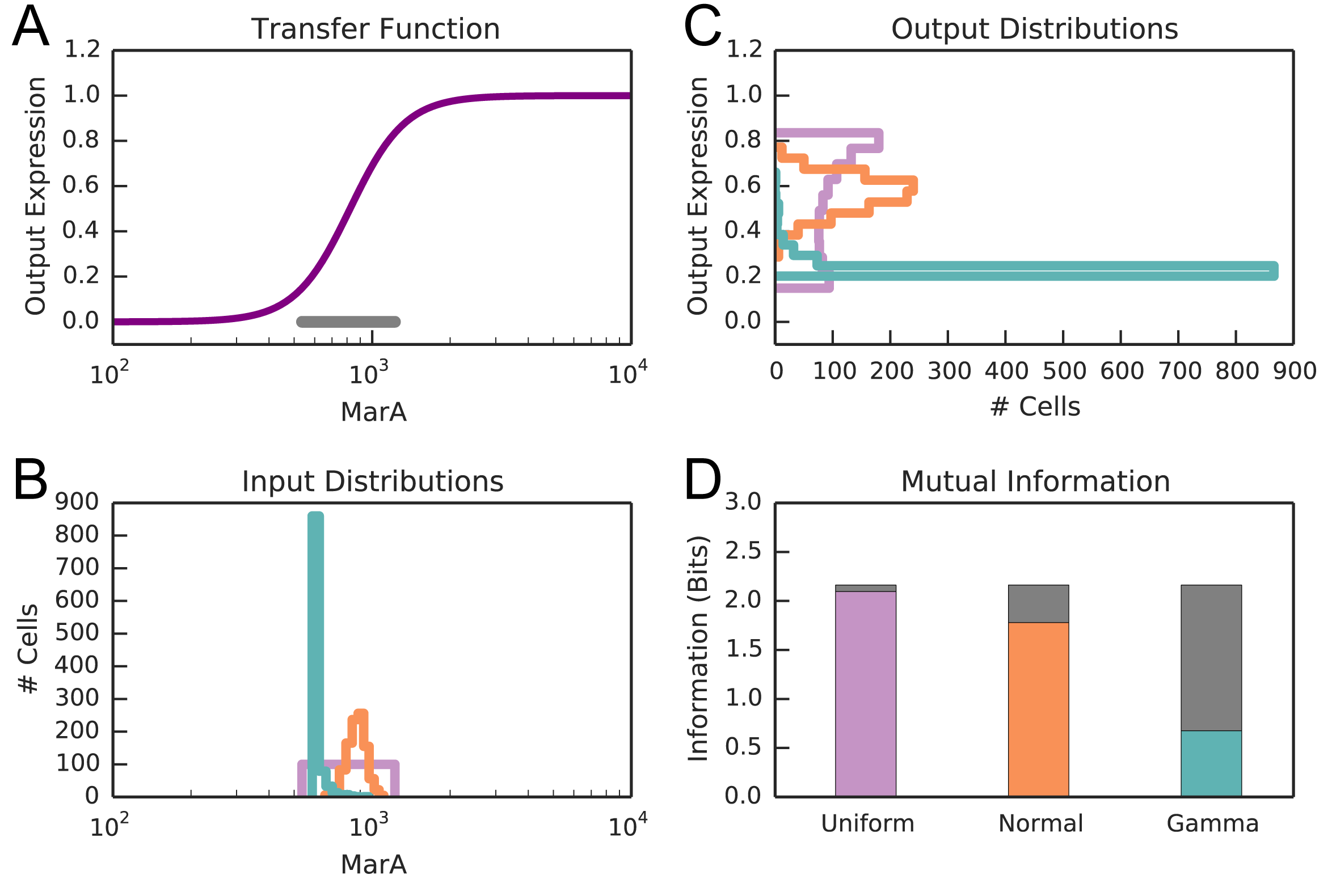

Supplement: S6 Fig — (A) Transfer function of PmicF with grey bar indicating bounded region for channel capacity. (B) Three examples of input distributions. (C) Input distributions mapped through transfer function shown in (A). (D) Mutual information as a fraction of channel capacity for each of the input distributions. Grey bars are the bounded channel capacity, which are the same for all, while the colored bars are the calculated mutual information for each of the input distributions and their mapped outputs. (TIF) [file pcbi.1005310.s007.tif]
